# Induction of antimicrobial peptides secretion by IL-1β enhances human amniotic membrane for regenerative medicine

**DOI:** 10.1038/s41598-017-17210-7

**Published:** 2017-12-05

**Authors:** Fatemeh A. Tehrani, Khashayar Modaresifar, Sara Azizian, Hassan Niknejad

**Affiliations:** 1 Department of Pharmacology, School of Medicine, Shahid Beheshti University of Medical Sciences, Tehran, Iran; 2 Department of Biomaterials, Faculty of Biomedical Engineering, Amirkabir University of Technology, Tehran, Iran

## Abstract

Due to antibacterial characteristic, amnion has been frequently used in different clinical situations. Developing an *in vitro* method to augment endogenous antibacterial ingredient of amniotic epithelial and mesenchymal stem cells is desirable for a higher efficacy of this promising biomaterial. In this study, epithelial or mesenchymal side dependent effect of amniotic membrane (AM) on antibacterial activity against some laboratory and clinical isolated strains was investigated by modified disk diffusion method and colony count assay. The effect of exposure to IL-1β in production and release of antibacterial ingredients was investigated by ELISA assay. The results showed that there is no significant difference between epithelial and mesenchymal sides of amnion in inhibition of bacterial growth. Although the results of disk diffusion showed that the AM inhibitory effect depends on bacterial genus and strain, colony count assay showed that the extract of AM inhibits all investigated bacterial strains. The exposure of AM to IL-1β leads to a higher level of antibacterial peptides secretion including elafin, HBD-2, HBD-3 and cathelicidic LL-37. Based on these results, amniotic cells possess antibacterial activity which can be augmented by inflammatory signal inducers; a process which make amnion and its epithelial and mesenchymal stem cells more suitable for tissue engineering and regenerative medicine.

## Introduction

Extra-embryonic membrane is one of the most frequently used sources for regenerative medicine. Amnion is the innermost part of this membrane which has five layers^1^. The amnion consists two distinct cell population; Epithelial cells and Mesenchymal cells with characteristics of stem cells^2^. The amniotic epithelial cells contain non-intestinal-type surface microvilli, intracytoplasmic lumina lined with microvilli, and intercellular junctions; and amniotic mesenchymal cells have rough endoplasmic reticulum profiles, lipid droplets, and well-developed foci of contractile filaments with dense bodies^3^. Furthermore, the extracellular matrix of amnion (which mostly secreted by amniotic epithelial and mesenchymal cells) in the basement membrane and compact layer contains collagen, laminin^1^, elastin, fibronectin, vitronectin, nidogen^4^, and hyaluronic acid^5^, which are important for using the AM as a natural scaffold in tissue engineering and regenerative medicine^1,6,7^.

The amniotic membrane (AM) possesses different characteristics including low immunogenicity^8^, anti-inflammatory effect^9^, angiogenic modulatory properties^10,11^, anti-cancer properties^12^ and antimicrobial activity^13^; which make it suitable for clinical situations such as ophthalmology^14^.

Amnion antimicrobial activity is caused by peptides such as defensins, SLPI (Secretory Leukocyte Protease Inhibitor), and elafin, which are often displayed in mucosal surface and synthesized by amniotic epithelial and mesenchymal cells^15^. Natural antibacterial peptides are necessary for innate immune system which protect pregnancy against bacterial, fungal and viral infections^16,17^. Maternal infection during the pregnancy and within delivery can cause preterm delivery and disorders or diseases in fetal and newborn^18^. To prevent the effect of contaminations during pregnancy and delivery, uterus, endometrium^19^, placenta and fetal membranes secrete natural antimicrobial peptides^20^.

Human amnion is one of the tissues which produces and secretes antimicrobial peptides. Human defensins are the first group of these peptides which are secreted in mucosal tissues^21^. Human beta-defensin 2 and 3 display regularly in the amniotic cells^22^ by being exposed to the Gram negative and Gram positive bacteria^23^. Elafin is the other amnion antimicrobial peptide that act as anti-protease^20,24^ and elastase inhibitor^25,26^. Additionally, the AM possesses some natural elements like hyaluronic acid^5^, lactoferrin, Cathelicidin, and interleukin-1 receptor antagonist that decrease inflammation and bacterial infection^27,28^.

The main aim of this article was to study the epithelial and mesenchymal side dependent antibacterial effect of fresh amnion on different bacterial strains. Moreover, this study focused on secretory state of amniotic cell ingredients which can be induced by inflammatory stimuli such as IL-1β or bacterial LPS; a procedure which boosts antimicrobial capability of amniotic membrane in regenerative medicine.

## Materials and Methods

### Amnion preparation

All Methods and experimental procedures of this study were in accordance with the experimental guidelines and regulations and approved by the research ethic committee of Shahid Beheshti University of Medical Sciences. Human placenta (n = 30) was collected immediately after the elective Caesarean delivery under sterile conditions and treated with cold normal saline to be transferred to laboratory. Placentas were obtained from healthy women at gestational ages 36–38 weeks with serologically negative results for HIV, HBV, HCV and syphilis. Informed consent was obtained from the parents.

Amnion was separated from the chorion by peeling in sterile condition under laminar flow. Then amnion was washed with cold phosphate buffered saline (PBS) three times to remove blood residue.

### Microbial assay

Antibacterial property of amnion was measured by modified disk diffusion method. This method was adapted from the Clinical and Laboratory Standard Institute (CLSI) protocol. *Pseudomonas aeruginosa* ATCC 27853*, Staphylococcus aureus* ATCC 25923*, Escherichia coli* ATCC 25922 and two clinically isolated sensitive strains of *E. coli* (T3 and T4) were used for antibacterial susceptibility test.

All bacterial strains were cultured on the blood agar medium (Merck, Germany). After being incubated overnight at 35 ± 2 °C, some colonies were harvested from plates and separately suspended in the sterile normal saline. The turbidity was equivalent to a 0.5 McFarland standard. Finally, these bacteria were cultured on the Muller-Hinton agar plates (Merck, Germany).

After being isolated and washed, the fresh amnion was cut into small pieces (1 × 1 cm). Each of the pieces were placed on the seeded Muller-Hinton agar plate in 20–30 minutes after separation, according to the modified disk diffusion method^13,29^. Amnion pieces were put separately in two ways, epithelial side up and mesenchymal side up as an antibacterial peptide releasing disk. Then the plates were incubated at 35 ± 2 °C overnight and the inhibitory effects were measured.

In order to avoid probable contamination within processing and preparation of the AM, a small piece (1 × 1 cm) of fresh AM was put on the Muller-Hinton agar plates and microbial growth was controlled 24 h after cultivation of tissues.

### Amnion extract antibacterial effect

Antibacterial effect of amnion extract was examined with a modified method in which a piece of amnion (1 × 1 cm) was cut into smaller sections and added to equivalent volume of PBS. The extract was obtained by sonicating the amnion on ice for 10 minutes with 80 W and 0.5 s cycle (Hielscher, Ultrasound Technology, Germany). Then membrane residues were removed by centrifuging at 800 rpm for 4 min. 500 μl of amnion extract was added to the surface of each Muller-Hinton agar plates. After drying the surface of plates, the bacterial inoculum was added to them. The number of colonies was counted after overnight incubation at 35 ± 2 °C.

### Evaluating the secretory state of natural antimicrobial peptides

To evaluate secretory state of antimicrobial agents from amniotic cells, the amniotic membrane was cut into 12 small pieces that were each 2 × 2 cm, and the pieces were individually cultured in a 12-well plate. To each well, 1 mL of DMEM/F12 containing FBS 10% and penicillin-streptomycin 1% was added. The plates were incubated in 37 °C with 5% CO2.

After 24 hours, medium was changed to serum-depleted (1% FBS) for 20 hours before treatments were added; 1% FBS was used because our previous study demonstrated decreased amniotic cell viability after 24 hours of culture in the absence of serum^30^. Half of the plates were treated with 10 ng/mL recombinant human interleukin (IL)−1beta (Peprotech, London, UK) to induce production and secretion of natural antimicrobial. The plates were incubated in 37 °C with 5% CO2 for 24 h, after which the supernatant was collected from each well.

### Enzyme-linked immunosorbent assay

Elafin, HBD-2, HBD-3 and cathelicidin LL-37 concentrations in the supernatant of amniotic membrane cultures were determined by elafin ELISA kit (Abcam, USA), HBD-2 assay kit (PeproTech) and HBD-3 and Cathelicidin LL-37 ELISA kits (HyCult Biotech), according to company instructions.

### Statistical analysis

Statistical comparisons were performed as mean ± standard error of the mean (SEM). Statistical significance was determined by means of one-way analysis of variance (ANOVA) followed by Tukey’s post-test. A P-value less than 0.05 was considered statistically significant.

### Data availability

All data generated or analyzed during this study are included in this published article.

## Results

Possible bacterial contaminations of membranes were controlled by culturing the fresh tissues on Muller-Hinton agar plates and microbiologically proper samples without bacterial contaminations were used for data interpretation. Totally, 30 amnion were examined in this study and at least 6 amnion were used for each bacterial strain in epithelial or mesenchymal sides separately. In the statistical analysis, only the groups with 50% inhibition zone in cultured plates were used.

Based on the disk diffusion method, the inhibitory effect of the AM was appeared in *P. aeruginosa* ATCC 27853*, E. coli* T3 and T4 after 24 hours of incubation (Table 1).

**Table 1:** The inhibitory effects of epithelial side up and mesenchymal side up amnion on three bacterial strains based on the disk diffusion method.

Bacterial strain	Amnion							
	**Epithelial side up**				**Mesenchymal side up**			
*P. aeruginosa* ATCC 27853	Inhibition zone (n = 14)^a^	Elimination under tissue (n = 14)^b^	Mean inhibition zone (n = 14)	Max. inhibition zone	Inhibition zone (n = 14)^a^	Elimination under tissue (n = 14)^b^	Mean inhibition zone (n = 14)	Max. inhibition zone
	12^c^, 2^d^	14	2.8 mm	5 mm	14	13^c^, 1^d^	2 mm	4 mm
*E. coli* T3	Inhibition zone (n = 21)^a^	Elimination under tissue (n = 19)^b^	Mean inhibition zone (n = 21)	Max. inhibition zone	Inhibition zone (n = 17)^a^	Elimination under tissue (n = 17)^b^	Mean inhibition zone (n = 21)	Max. inhibition zone
	19^c^, 2^d^	14^c^, 5^d^	1.2 mm	2 mm	16^c^, 1^d^	14^c^, 3^d^	1 mm	1 mm
*E. coli* T4	Inhibition zone (n = 14)	Elimination under tissue (n = 15)	Mean inhibition zone (n = 14)	Max. inhibition zone	Inhibition zone (n = 22)^a^	Elimination under tissue (n = 14)^b^	Mean inhibition zone (n = 14)	Max. inhibition zone
	12^c^, 2^d^	12^c^, 3^d^	1.3 mm	2 mm	19^c^, 3^d^	12^c^, 2^d^	1 mm	2 mm

^a^The number of plates examined for inhibition zone.

^b^The number of plates examined for elimination of bacterial growth under tissue.

^c^The number of plate with inhibition zone or elimination.

^d^The number of plate without inhibition zone or elimination.

**Figure 1:**
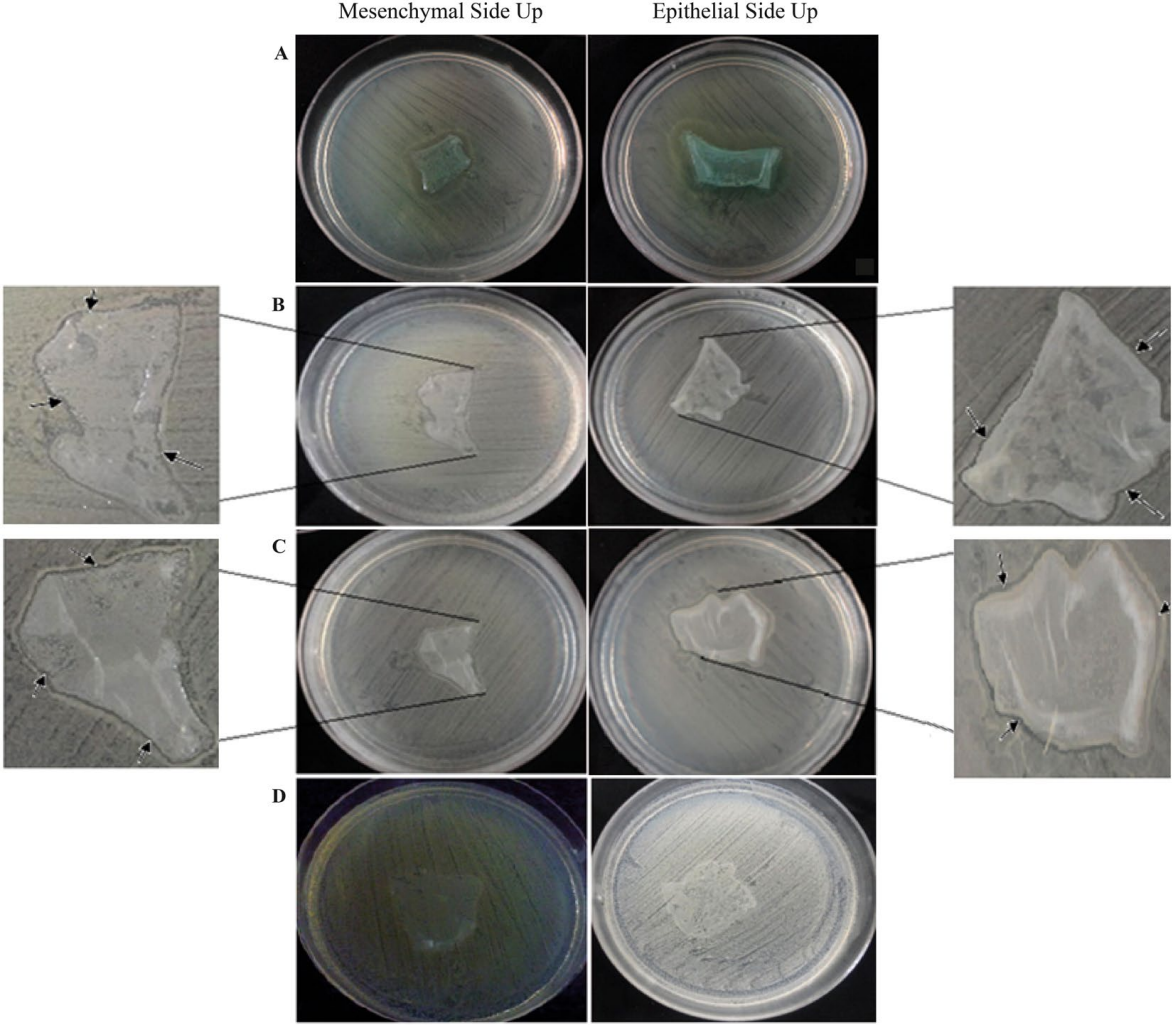
The inhibition zone of mesenchymal side up and epithelial side up amnion in, (**a**) *P. aeruginosa* ATCC 27853 culture, (**b**) *E. coli T3* culture (The arrows indicate the inhibition zone), (**c**) *E. coli* T4 culture (The arrows indicate the inhibition zone), (**d**) No inhibitory effect appeared in; *E. coli* ATCC 25922 (left) and *S. aureus* ATCC 25923 (right). The experiment was performed in triplicate (n = 5 for each time).

The growth was decreased under and in the edge of the tissues in both epithelial side and mesenchymal side up amnion. The mean of inhibition zone was about 2 mm in the epithelial side up AM and 2.8 mm in mesenchymal side up AM for *P. aeruginosa* ATCC 27853 cultures. The maximum inhibition (5 mm) was seen in this group (Fig. 1a) (Table 1).

The inhibitory effect among the *E. coli* strains demonstrated different results. The inhibition zone was appeared in two sensitive clinical isolated *E. coli* (T3 and T4) in both epithelial and mesenchymal side up. Figure 1b and Fig. 1c demonstrate the inhibition zone in *E. coli* T3 and *E. coli* T4, respectively.

**Figure 2:**
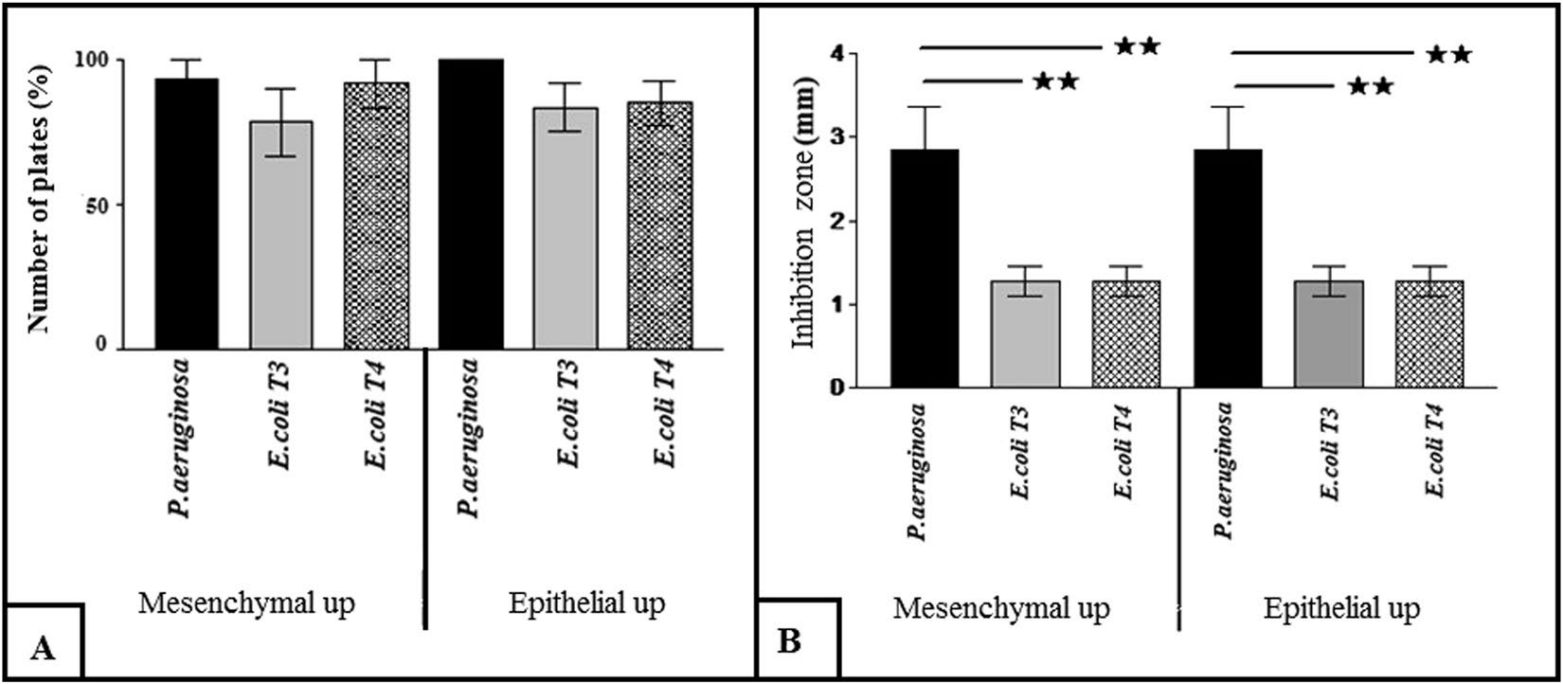
(**a**) The number of plates (%) with inhibition zone in bacterial culture in epithelial side up and mesenchymal side up amnion; (**b**) The mean of inhibition zone (mm) in epithelial side up and mesenchymal side up amnion in bacterial culture after overnight incubation (P < 0.01★★). The experiment was performed in quadruplicate (n = 5 for each time).

Statistical analysis showed that there is no significant difference in the number of plates in which the inhibitory effect was seen against *P. aeruginosa* ATCC 27853, *E. coli* T3 and T4 (Fig. 2). However, the inhibition zone in *P. aeruginosa* ATCC 27853 is significantly (P < 0.01) greater than two clinical isolated *E. coli* T3 and T4 (Fig. 2).

To investigate the effects of amnion side on antibacterial activity, we compared the results between mesenchymal and epithelial sides of amnion. As shown in Fig. 2, there is no significant difference between the number of plates and inhibition zone of *P. aeruginosa* ATCC 27853 which incubated with mesenchymal side amnion in comparison to those of incubated with epithelial side amnion. The same results were achieved from incubation of *E. coli* T3 and T4 with both mesenchymal and epithelial sides of amnion.

No inhibitory effect was seen in *E. coli* ATCC 25922 *and S. aureus* ATCC 25923 after 24 hours of incubation under and in the edge of both epithelial side up and mesenchymal side up amnion (Fig. 1d).

**Figure 3:**
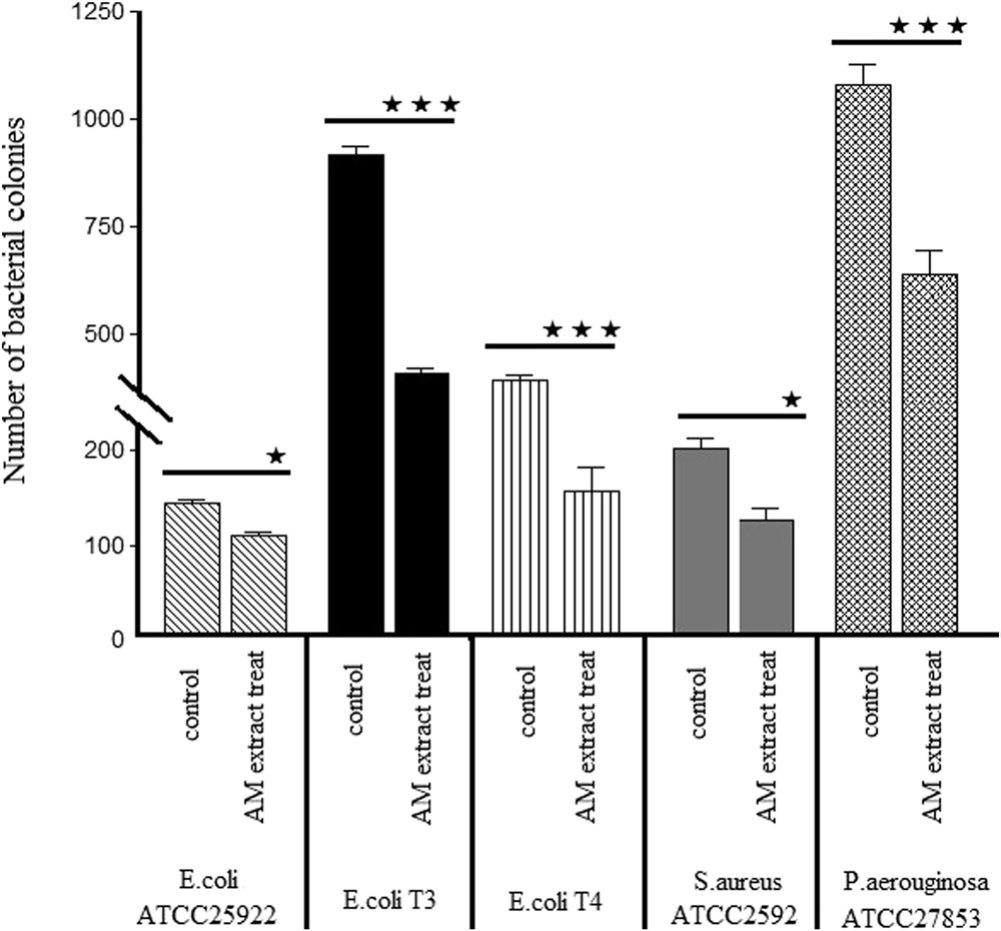
The number of bacterial colonies appeared in Muller-Hinton agar plates treated with amnion extract (P < 0.05 ★, P < 0.001 ★★★). The experiment was performed in quadruplicate (n = 5 for each time).

**Figure 4:**
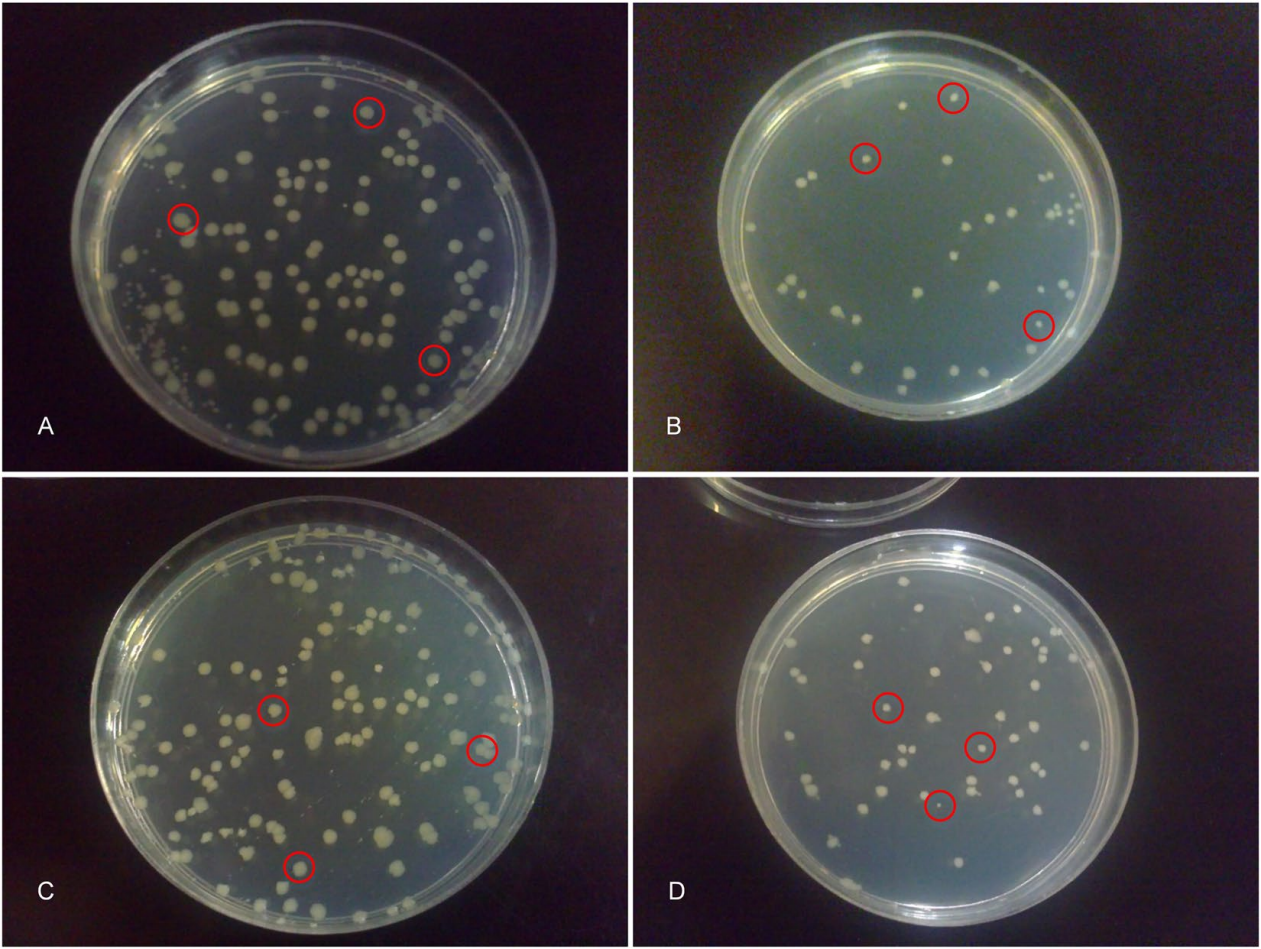
The extract of amnion decreased the size and number of (**a**) S. aureus ATCC 25923 and (**c**) *E. coli* ATCC25922 colonies compared to the control groups (**b** and **d**, respectively). The reduced size of colonies is shown by red circles. The experiment was performed in triplicate (n = 5 for each time).

In order to consider the effects of the contents of amniotic cells, the AMs were sonicated and the inhibitory effects of amnion extract were evaluated on *P. aeroginosa* ATCC 27853, *S. aureus* ATCC 25923, *E. coli* ATCC 25922, and two clinical sensitive strains of *E. coli* (T3, T4). Consistent with the results of disk diffusion method, the number of colonies decreased in *P. aeroginosa* ATCC 27853 and two clinical sensitive strains of *E. coli* (T3, T4) significantly in comparison to control groups (Fig. 3). Surprisingly, the extract of amnion significantly reduced the number of colonies of S. aureus ATCC 25923 and *E. coli* ATCC25922. In addition to the number of colonies, the extract of amnion decreased the size of S. aureus ATCC 25923 and *E. coli* ATCC25922 colonies compared to the control groups (Fig. 4).

To evaluate secretory state of amniotic cells after induction with IL-1β, elafin, HBD-2, HBD-3 and cathelicidin LL-37 protein levels were measured in amniotic membrane culture supernatant by ELISA. The rational for choosing IL-1 beta as pro-inflammatory cytokine was a pilot study in which we compared 10 ng/ml of TNF-alpha with the same concentration of IL-1 beta in production of HBD-3 at time points 6, 12, 24 and 48 hours. Although the primary results showed that both pro-inflammatory cytokines significantly up-regulated HBD-3 production compared to unstimulated control, HBD-3 production was significantly higher at each time point in response to IL-1 beta (data not shown). As evaluated by trypan blue exclusion, treatments with IL-1β had no significant effect on amniotic cell numbers or viability after 24 hours.

**Figure 5:**
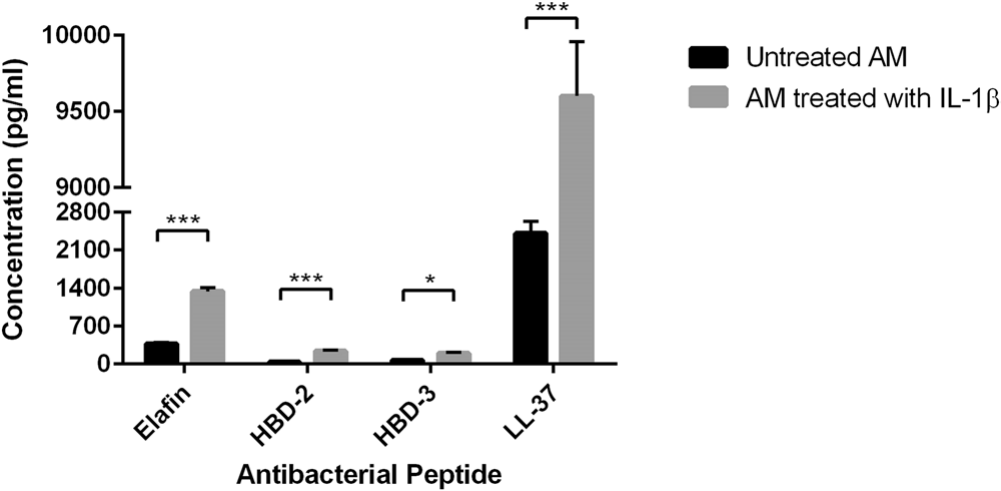
Antibacterial peptides concentration in the AM culture supernatant before and after treatment with IL-1β for 24 hours (P < 0.05 ★, P < 0.001 ★★★). The experiment was performed in quadruplicate (n = 5 for each time).

As shown in Fig. 5, amniotic membrane releases a basal level of antimicrobial peptides elafin, HBD-2, HBD-3 and cathelicidin LL-37 in the culture medium. Significantly higher levels of elafin, HBD-2, HBD-3 and cathelicidin LL-37 proteins were secreted by the membranes treated with IL-1β 10 ng/mL for 24 hours.

## Discussion

In this study, the antibacterial property of amnion was examined on different bacterial strains and the side dependent effect of amniotic membrane was also investigated on antibacterial activity. Furthermore, the amnion extract was used to optimize the inhibitory effect.

Disk diffusion is a commonly used method to evaluate antibacterial property of the AM. Talmi *et al*. used the modified disk diffusion method for investigating the amniotic membrane antimicrobial activity on coagulase positive *staphylococcus*, *E. coli, Kelebsiella pneumonias, P. aeruginosa, Proteus mirabilis*. They demonstrated that the inhibition zone appears exactly under the tissue due to the close contact between amnion and culture plate^13^. In further studies, Kjaegaarda *et al*. flattened the fresh amnion on the nitrocellulose filter and utilized it *in vitro* study. They reported that a narrow inhibition zone (1 mm) appeared in Group A streptococcus, *Staphylococcus saprophyticus*, *E. coli, P. aeruginosa* and *S. aureus* ^31^. We also previously demonstrated the antibacterial properties of cryopreserved AM by this method^32^.

In the present study, we used both standard and clinical bacterial strains to investigate antibacterial property by disk diffusion method. In accordance to the results of disk diffusion method, the inhibitory effect of fresh amnion appeared in *P. aeruginosa* and two clinical isolated strains of *E. coli* (T3 and T4) opposed to *S. aureus* ATCC 25923 and *E. coli* ATCC 25922. The results of disk diffusion method showed that the amnion inhibitory effect was dependent on bacterial genus and bacterial strain. According to our results, it seems that the inhibitory effect in disk diffusion method is due to either the contact between the amnion and bacterial strains^13^ or achieving a secretion threshold of antibacterial ingredients which is enough to inhibit the bacterial activity of some strains. For instance, elafin can inhibit serine peptidase that is a virulence factor in *P. aeruginosa* in a critical threshold concentration which is not enough to inhibit growth of the other bacterial strains^33,34^. On the other hand, using the amnion extract reduced the size and number of colonies in all strains. In the case of two strains which were not inhibited in disk diffusion method but inhibited using the amnion extract (*S. aureus* ATCC 25923 and *E. coli* ATCC 25922), we have two options. Either amniotic membrane cannot release its ingredients in disk diffusion condition or the amniotic antibacterial ingredients are non-secretory. As shown in results, exposing the AM to IL-1β resulted in a higher amount of elafin, HBD-2, and HBD-3 and cathelicidin LL-37 secretion which confirmed secretory state of amniotic antibacterial ingredients. This result is consistent with previous study in which we showed that AM releases some anti-cancer and immune-modulatory agents in condition medium^35,36^. Therefore, the non-secretory state of amniotic antibacterial ingredients is not acceptable and it seems that the disk diffusion method does not provide an appropriate condition for amnion to display its antibacterial property. To the best of our knowledge, disk diffusion method is the most common method for assessing the AM antibacterial property; and since it is not a proper method for this aim, it is necessary to modify and develop alternative methods to attain a reliable method for investigating the antibacterial property of AM.

In this study the side dependent effect of amnion on bacterial activity was examined. There are no significant difference between epithelial side up and mesenchymal side up amnion in inhibitory effect. This similarity in two sides of amnion suggests that not only amniotic epithelial cells cause inhibitory effect, but also amnion mesenchymal cells can display antibacterial property. Although all previous studies focused on antibacterial properties of amniotic epithelial cells, amniotic mesenchymal cells also could have antibacterial characteristics^32,37^. Mesenchymal stem cells have antibacterial characteristics. It has been shown that lipocalin 2^38^, beta-defensin 2^39^ and LL-37 (human cathelicidin antimicrobial peptide, hCAP-18)^40^ in mesenchymal stem cells from the other sources such as bone marrow and adipose demonstrate antibacterial property against bacterial strains; hence, the amniotic mesenchymal cells also may contribute to inhibitory effect of amniotic membrane. More studies would be required to determine the antibacterial effect of amniotic mesenchymal cells.
